# In Vitro Antioxidant, Anti-Biofilm, and Solar Protection Activities of *Melocactus zehntneri* (Britton & Rose) Pulp Extract

**DOI:** 10.3390/antiox8100439

**Published:** 2019-10-01

**Authors:** Verônica Giuliani de Queiroz Aquino-Martins, Luciana Fentanes Moura de Melo, Larissa Marina Pereira Silva, Thales Rodrigo Targino de Lima, Moacir Fernandes Queiroz, Rony Lucas Silva Viana, Silvana Maria Zucolotto, Vania Sousa Andrade, Hugo Alexandre Oliveira Rocha, Katia Castanho Scortecci

**Affiliations:** 1 Pós-Graduação em Bioquímica, Centro de Biociências, Universidade Federal do Rio Grande do Norte, Natal CEP 59078-970, Brazil; verinhaquino@hotmail.com (V.G.d.Q.A-M.); lucianafentanes@gmail.com (L.F.M.d.M.); moacirfqn@gmail.com (M.F.Q.); hugo-alexandre@uol.com.br (H.A.O.R.); 2 Laboratório de Transformação de Planta e Análise em Microscopia (LTPAM), Departamento de Biologia Celular e Genética, Centro de Biociências, Universidade Federal do Rio Grande do Norte, Natal CEP 59078-970, Brazil; 3 Laboratório de Biotecnologia de Polímeros Naturais (BIOPOL), Departamento de Bioquímica, Centro de Biociências, Universidade Federal do Rio Grande do Norte, Natal CEP 59078-970, Brazil; 4 Laboratório de Produtos Naturais e Bioativos (PNBio), Departamento de Farmácia, UFRN, Natal CEP 59078-970, Brazil; larissamarinaps@gmail.com (L.M.P.S.); silvanazucolotto@ufrnet.br (S.M.Z.); 5 Laboratório de Ensaios Antimicrobianos e de Citotoxicidades (LEAC), Departamento Microbiologia e Parasitologia, Centro de Biociências, Universidade Federal do Rio Grande do Norte, Natal CEP 59078-970, Brazil; thalesdlima@hotmail.com (T.R.T.d.L.); vaniasandrade@gmail.com (V.S.A.)

**Keywords:** coroa-de-frade, antioxidant activity, MTT reduction, SPF

## Abstract

Cactaceae plants are important due to their nutritional and therapeutic values. This study aimed to identify the phytochemical profile and biological activities of six *Melocactus zehntneri* pulp extracts: hexane extract (HE), chloroform extract (CE), ethanol extract (EE), methanol extract (ME), final water extract (FWE), and water extract (WE). Sugar, phenolic compounds, and protein content of the extracts were determined. Then thin layer chromatography (TLC) was performed to detect the presence of terpenes (ursolic and oleanolic acids), saponins, sugars, and glycoproteins. These extracts were analyzed for antioxidant activity via in vitro assay. HE showed 75% ferric chelating activity. All extracts showed 80–100% superoxide and hydroxyl radical-scavenging activities, respectively. Further, all extracts at 25 µg/mL showed 60% activity against DPPH. Moreover, in the 3T3 cells lines, no cytotoxicity was observed; however, therapeutic activity against the effects of the H_2_O_2_ treatment was exhibited. Finally, the polar extracts (EE, ME, FWE, and WE), particularly WE, elicited activity against the biofilms of *Staphylococcus epidermidis*, and HE and CE expressed a capacity for solar protection.

## 1. Introduction

Oxidative stress has been associated with various neurodegenerative diseases such as Alzheimer’s and Parkinson’s disease, inflammatory diseases, type 2 diabetes, cardiovascular diseases, aging-related diseases, and tumors [1,2,3]. Oxidative stress has also been correlated with Reactive Oxygen Species (ROS) imbalance, which may promote macromolecular damage in lipids, proteins, and DNA molecules, leading to loss of cell integrity and function [2,4]. Cells have different proteins (cell machinery) that maintain cell homeostasis, and antioxidant molecules may be isolated from vegetable and fruit sources [5,6].

Plants produce a mixture of compounds known as secondary metabolites, which are usually divided into three main categories: terpenes, phenolic compounds, and alkaloids. These metabolites exhibit a variety of biological and pharmacological properties; thus, identification and isolation of compounds from these complex mixtures, as well as their use as daily foods, are important research topics in this field [5,7,8,9].

Cactaceae plants are known to be composed of considerable amounts of water, sugars, and proteins. These plants can be found in desert regions expanding from Southern areas of the United States, the Andes region, Mexico, and northeast Brazil. Usually, these plants grow in regions with a semiarid climate, warm and dry conditions, and low rainfall [10]. *Melocactus zehntneri* (Britton & Rose) Luetzelburg, also known as crown-of-monk (coroa-de-frade), is restricted to the Northeast region of Brazil.

*Melocactus* is identified by the cephalium (a red crown at the tip of the cactus) [11]. This plant is globe-shaped, elongated, unbranched, with whole ribs, cylindrical and curved spines, with adventitious roots that emerge from the base of the cladodium (modified-stem). Their internal mucilaginous constitution is one of the adaptive characteristics of this group of plants to the xeric environment [12].

Plants of the genus *Melocactus* are characterized by a complex mixture of essential oils, flavonoids, steroids, terpenoids, alkaloids, carbohydrates, and amino acids [13]. *M. zehntneri* has been used in traditional medicine to treat respiratory conditions, influenza, physical fragility, throat inflammation, whooping cough, and in “uterine cleaning”. Furthermore, this cactus is also used as an exotic food for making sweets, an animal food in the dry season, and for ornamental use in gardens [13]. However, few scientific studies have focused on the use of this plant in popular medicine. This study aimed to determine the phytochemical composition of this plant via thin-layer chromatography (TLC). The phytochemical composition is related to chemical compounds produced by plants and correlated to certain biological activities. Furthermore, the in vitro antioxidant activity, cell viability, effects on biofilm production, and in vitro solar protection factor (SPF) were also evaluated. Moreover, due to increasing resistance in Brazil acute care units; the biofilm production was evaluated for *S. epidermidis* 70D (methicillin resistant *S. epidermidis*, MRSE), which is considered an opportunistic pathogen responsible for chronic infections. These bacteria have the ability to form biofilms on indwelling medical devices of hospitalized patients. Moreover, this pathogen is resistant to antimicrobials used in medicinal therapies [14].

## 2. Materials and Methods 

### 2.1. Plant Material

*Melocactus zehntneri* cephalium were collected at Lajes/RN, Brazil on August 2017, (5°38’41,912″ S and 36°12’42,726″ W) in the Caatinga biome, a region with water precipitation ≤ 2 mm, temperature maximum 34 °C and minimum 22 °C, ultraviolet index UV = 10 [15]. Dr. Leonardo de Melo Versieux identified one specimen and it was deposited at the UFRN herbarium with voucher n° 23796. The research was registered at SISBIO n° 54214. 

### 2.2. Pulp Extract Preparation

In order to obtain the different extracts, the part inside of the cladode, called the pulp, was isolated. This corresponds to the modified globe-shaped stem. Fresh pulps from six cacti adults were weighted (8823 g), then lyophilized (using Liotop-L202, Prismalab, Rio de Janeiro, Brazil) and re-weighted after drying (300 g) to determine the amount of solvent used in the serial extraction following the ratio 1:10 (pulp *w*/solvent *v*). The lyophilized pulp was transferred to an Erlenmeyer flask using solvents (Synth, Brazil) in the following order: hexane, chloroform, ethanol, methanol, and distilled water. In addition, one extraction was performed with only distilled water (WE). This material was kept at 50 rpm (Tecnal Shaker, Tecnal, Piracicaba, Brazil) for 24 h in a room that has the temperature at 23–24 °C controlled with air conditioner. The samples during the extraction process were protected from light by covering with aluminum foil and it was done in a refrigerated room at 24 °C. The extract was filtered using Whatman n° 1 paper and the next solvent was added to the cactus pulps, under the same conditions. The filtered extracts were concentrated up to 25 mL using a rotavapor (Tecnal-TE 210, Tecnal, Piracicaba, Brazil) at 40 °C and then lyophilized in order to obtain a powder that was kept in conical tubes (2 mL) at 18 °C until use. The extracts obtained were: HE (Hexane Extract), CE (Chloroform Extract), EE (Ethanol Extract), ME (Methanol Extract), FWE (Final Water Extract), and WE (Water Extract). 

### 2.3. Total Content of Phenolic Compounds, Sugar, and Protein

Total phenolic content was determined according Folin–Ciocalteau’s colorimetric method using gallic acid (Sigma-Aldrich, Saint Louis, MO, USA) as a standard, at 765 nm [16]. Total sugars were estimated by phenol-H_2_SO_4_ using D-glucose (Sigma-Aldrich, Saint Louis, MO, USA) as standard, at 490 nm [17]. Total protein content was estimated by the Bradford method using bovine serum albumin as standard (Sigma-Aldrich, Saint Louis, MO, USA) at 595 nm [18]. All readings were taken using a spectrophotometer (Biotek Epoch Microplate, California, CA, USA). 

#### 2.3.1. Phytochemistry Screening by Thin Layer Chromatography (TLC)

TLC assay was performed in triplicates and aluminum chromatoplates covered by silica gel 60 GF_254_ (Merck) were used as stationary phase in a saturated chamber [19]. All extracts were tested in the mobile phase and standards were applied at the same concentrations 10 mg/mL. Chromatograms were obtained using different mobile phases in order to determine major secondary metabolites: (I) toluene/ethyl acetate/formic acid (5:5:0.5—*v*/*v*/*v*); (II) ethyl acetate/formic acid/water/methanol (10:1.6:1.5:0.6—*v*/*v*/*v*/*v*); ethyl acetate/formic acid/water/methanol (10:0.5:0.2:0.6—*v*/*v*/*v*/*v*); ethyl acetate/formic acid/water/methanol (8:1.6:2.0:0.6—*v*/*v*/*v*/*v*), and ethyl acetate/formic acid/water/methanol (8:1.8:2.0:0.6—*v*/*v*/*v*/*v*); (III) propanol/water/ethyl acetate/formic acid (4:0.5:0.5:0.5—*v*/*v*/*v*/*v*) and (4:0.7:0.5:0.8—*v*/*v*/*v*/*v*); (IV) methanol/chloroform (5:5—*v*/*v*). The developer reagents used were: sulfuric vanillin, Natural Reagent A (difenilboriloxietilamina) 0.5%, iodine, timol, Dragendorff Reagent, Lieberman, anisaldeide, ferric chloride, and ninhydrin. The reference standards used were diogenin, saponin, ursolic acid, oleanolic acid, and sugars: raffinose, fructose, mannitol, sucrose, galactose and lactose. All standards were purchased from Sigma-Aldrich Saint Louis, MO, USA). The plates were dried and visualized under UV light at 254 nm and when using Natural Reagent A, at 365 nm. 

#### 2.3.2. Cellulose Descendant Thin Layer Chromatography

TLC-Cellulose was performed using a sheet of 1.00 × 0.5 m cellulose paper in a pre-saturated cell. A system with a solution of ethyl-acetate/pyridine/distilled water (8:2:1) was used as the mobile phase. The monosaccharides galactose, fructose, glucose, mannose, rhamnose, and xylose, and uronic and glucuronic acids were used as standards (Sigma). Chromatograms were developed with a silver nitrate solution (3.3% *w*/*v*) [19] with modifications. Samples and standards were used at the same concentrations—10 mg/mL.

### 2.4. In Vitro Antioxidant Activity

Different in vitro assays were performed in order to evaluate the antioxidant activities under different conditions, as previously described by Melo-Silveira et al. [20]. The assays were performed in triplicates and each experiment was also repeated three times. All readings were done using a spectrophotometer (Biotek Epoch Microplate, California, CA, USA).

#### 2.4.1. Total Antioxidant Capacity (TAC)

This assay is based on the reduction of Mo^+6^ to Mo^+5^ by the extracts and subsequent formation of a green phosphate/Mo^+5^ complex at acidic pH. Extracts were transferred to different tubes at the concentrations of 100, 250, and 500 μg/mL and the reagent solution (0.6 M sulfuric acid, 28 mM sodium phosphate, and 4 mM ammonium molybdate). The extract and reagent solution were incubated at 95 °C for 90 min. [21]. Thereafter, these tubes were kept at room temperature to cool and then absorbance was measured at 695 nm using a spectrophotometer against a blank, consisting of all reagents in distilled water. The antioxidant capacity was expressed as ascorbic acid equivalents (EEA/g) used as standard. 

#### 2.4.2. Hydroxyl Radical Scavenging Activity

The hydroxyl radical scavenging activity was based on Fenton’s reaction (Fe^2+^ + H_2_O_2_ → Fe^3+^ + OH^−^ + OH•), as described by Smirnoff & Cumbes [22] with modifications. The extracts were evaluated in three concentrations (100, 250, and 500 μg/mL). Then, to the extracts 3 mL sodium phosphate buffer (150 mM pH 7.4) containing 10 mM FeSO_4_ × 7H_2_O, 10 mM EDTA, 2 mM sodium salicylate, 30% H_2_O_2_ (200 mL) was added, leading to the formation of the hydroxyl radical. The solutions were incubated at 37 °C for 1 h, and absorbance at 510 nm was measured, indicating the presence of hydroxyl radicals. Results were expressed as percentage of scavenging comparing to the standard gallic acid.

#### 2.4.3. Ferric Chelating 

The ferrous ion chelating capacity of the extracts was investigated according to the protocol described by Wang et al. [23]. In a solution of 5 mM ferrozine and ferric chloride (2 mM FeCl_2_), the samples were added at concentrations 100, 250, and 500 μg/mL and tubes were shaken for 10 min at room temperature, before measuring absorbance at 562 nm against a blank of ultrapure water. Results were compared to the standard EDTA.

#### 2.4.4. Copper Chelating

The copper chelating activity of the samples was determined using pyrocatechol violet method according to Anton. [24]. The reaction mixture contained sodium acetate (50 mM) buffer, samples (100, 250, and 500 μg/mL), pyrocatechol violet (4 mM), and copper II sulfate pentahydrate 50 μg/mL). The solution was homogenized and the absorbance measured at 632 nm in a microplate reader. EDTA was used as standard.

The results were expressed as % chelating effect of copper ions using the following formula: % chelating effect = [A Control − (A Sample/A Control)] × 100.

Where A control: absorbance of the control tube and A sample: absorbance of the sample tube. The control used was the sodium acetate (50 mM) buffer.

#### 2.4.5. Superoxide Scavenging Activity

This assay was determined as described by Wang et al. [23]. It was based on the ability of the extracts to inhibit the photochemical reduction of tetrazolium (NBT) in the riboflavin-light-NBT system. Thus, a mixture of the extracts at different concentrations (100, 250, and 500 μg/mL), 50 mM phosphate buffer (pH 7.8), 65 mM methionine, 10 mM riboflavin, 0.5 mM EDTA and 0.375 mM NBT, and 10 mM riboflavin was exposed for 10 min to a fluorescent lamp. The change in the color to blue due to the production of the formazan compound was monitored by absorbance at 560 nm. EDTA was used as control and distilled water as blank. Every reaction was conducted inside a dissipating chamber of light, except the blank. Results were expressed as percent scavenging: 

Percentage of radicals scavenging = ([A control − A sample]/[A control − A blank]) × 100. Acontrol: absorbance of the control, Asample: absorbance of the sample, and Ablank: absorbance of the blank. 

#### 2.4.6. Reducing Power Test

The reducing power of the samples was quantified as described by Wang et al. [23] and Athukorala et al. [16]. To the reaction mixture (0.2 M phosphate buffer (pH 6.6) and potassium ferricyanide (1% *w*/*v*) the extracts at concentrations 100, 250, and 500 μg/mL were added, to a final volume of 4 mL. This mixture was incubated at 50 °C for 20 min. The reaction was terminated by addition of the trichloro acetic (TCA) solution (10% *w*/*v*) plus distilled water and ferric chloride (0.1% *w*/*v*). The absorbance was measured at 700 nm, using phosphate buffer as blank. The result was expressed as a percentage of the activity reported by 0.1 mg/mL ascorbic acid (standard—Sigma-Aldrich, Saint Louis, MO, USA).

#### 2.4.7. DPPH Free Radical Scavenging Activity

The antioxidant capacity of the samples was determined by the antioxidant activity according to the method of Shimada et al. [25]. The extracts at concentrations of 25, 50, and 100 μg/mL and 150 μM DPPH were mixed and allowed to stand at room temperature for 30 min. The absorbance was then measured at 517 nm. The DPPH free-radical scavenging activity was calculated as follows: scavenging activity: (%) = [1 − (A1/A0)] × 100%, where A0 is the absorbance of the control (DPPH), and A1 is the absorbance of the samples.

### 2.5. Biofilm Assays

#### 2.5.1. Microorganisms and Inoculum Preparation

The bacterial reference strain *Staphylococcus epidermidis* 70D MRSE (*Staphylococcus epidermidis* methicillin resistant) INCQS-Fio Cruz was used. The inoculum was prepared by adjusting the turbidity to a concentration of 0.5 of the Mc Farland scale (10^8^ CFU/mL).

#### 2.5.2. Determination of Minimum Inhibitory Concentration (MIC) and Determination of Minimum Bactericidal Concentration (MBC)

The quantitative methodology of microdilution in MH broth (Mueller Hinton) (Hymedia) was used [26]. The extracts were diluted serially from 100 to 0.39 mg/mL for extracts HE, EE, ME, and WE, and diluted from 50 to 0.19 mg/mL for extracts CE and FWE.

As a negative control (NC): an aqueous solution with 20 μL of the inoculum was used; to the positive control (PC), gentamicin (Hypopharma) at 40 mg/mL was added with MH culture medium and 20 μL of the inoculum. In the growth control (GC), only 20 μL of the inoculum was added. The microplate was incubated at 37 °C during 24 h to determine the MIC. All tests were performed in triplicate.

To determine the MIC, 20 μL of CTT (2,3,5-triphenyltetrazolium chloride) (Inlab) 0.5% solution was added to each well and incubated at 37 °C for 2 h to reveal bacterial growth. The readings were done as the lowest concentrations of extracts visually inhibiting microbial growth.

The bactericidal activity of the extracts was measured after reading the MIC by seeding the contents of the micro-titer wells into BHI agar. The best reading from the wells containing the extracts as well as PC, NC, and GC groups were chosen. Then, 10 μL of each was seeded in separate spots on petri dishes, and incubated at 37 °C for 24 h. The MBC was considered the lowest concentration that completely prevented microbial growth in BHI agar.

#### 2.5.3. Evaluation of Anti-Biofilm Activity of *M. zehntneri* Extracts against *S. epidermidis* 70D MRSE

The protocol was adapted from Stepanovic [27]. Twenty microliters of the *S. epidermidis* cultures was used at MIC. For the best biofilm development, the medium used was Tryptic Soy Broth (TSB) supplemented with 1% glucose, and 180 μL was added in a 96-well plate. Then 100 μL of each extract, at the MIC was used for the biofilm formation. The medium and inoculum were added simultaneously. Negative controls contained only with TSB broth, 1% glucose and positive controls contained the same medium and 20 μL of the inoculum. The plate was incubated at 36 °C for 24 h, and subsequently washed with 0.9% sterile saline solution, kept at 65 °C for 1 h for fixing and stained with 200 μL of violet crystal 15 min at room temperature. Finally, it was rinsed with distilled water, dried at room temperature, and then 200 μL of ethanol (95% PA) was added for 30 min. The absorbance was measured at 570 nm. The consolidated biofilm process differs only from the extract addition step, which was done 24 h after the biofilm had been consolidated. The results were expressed according to the following formula:

% Inhibition = 100 − [{A570 nm Extract/A570 nm Positive Control} × 100]

### 2.6. Cell Viability

#### 2.6.1. MTT Reduction Test

For this assay, the murine fibroblast cell line NIH/3T3 (ATCC CRL-1658TM, Manassas, VA, USA) was used. The 3T3 cell line was grown in 75 cm^2^ flasks with Dulbecco’s modified Eagle’s medium (DMEM-LGC) [28]. Cells were seeded into 96-well plates at a density of 5 × 10^3^ cells/well and allowed to attach overnight in 200 μL of medium with 10% fetal bovine serum (FBS) (LGC) at 37 °C, 5% CO_2_. After 24 h, *M. zehntneri* extracts were added at different concentrations: 50, 100, and 250 μg/mL for a period of 24 h at 37 °C and 5% CO_2_. After this time, extracts were removed and 100 μL of fresh medium and 10 μL of 12 mM MTT (3-(4,5-Dimethylthiazol-2-yl)-2,5-diphenyltetrazolium bromide) (Sigma-Aldrich, São Paulo, Brazil) dissolved in PBS were added. The cells were then incubated for 4 h at 37 °C and 5% CO_2_ and in order to solubilize the reduced MTT product it was added 100 μL of absolute ethyl alcohol (Sigma-Aldrich) to each well followed by thorough mixing. The absorbance at 570 nm was measured. The percentage of cell viability was calculated as:

Percentage of MTT reduction = (Abs of sample/Abs of control) × 100

#### 2.6.2. Cell Viability Test against Oxidative Stress with H_2_O_2_

First, hydrogen peroxide (Synth) molarity was quantified by measuring absorbance at 240 nm using 1% and 10% hydrogen peroxide. The values obtained were then applied in the adapted formula of Lambert–Beer: Absorbance = Optical Path × Ɛ × Molar Concentration; where optical path = 0.5; Ɛ240 = 43.6 cm.M^−1^, which is a correction factor. 

Once molar concentration was determined as 1.75, five concentrations of hydrogen peroxide 0.05, 0.1, 0.25, 0.5, and 1 were used to identify a concentration that promoted 50% MTT inhibition in the cells (IC_50_). Thus, the concentration of hydrogen peroxide was determined to be 0.1 M. Then, using the methodology described above in Section 2.6.1, and an extract concentration of 250 μg/mL, the effect of hydrogen peroxide on the 3T3 cell line was analyzed at 0.1 M H_2_O_2_ (IC _50_) for 24 h at 37 °C and 5% CO_2_. The H_2_O_2_ treatment was carried out using three approaches: preventive action—the *M. zehntneri* extracts were added 30 min before the addition of hydrogen peroxide; curative action—the extracts were added 30 min after the hydrogen peroxide addition; and concurrent action—the extracts were placed together with the hydrogen peroxide. After this period of treatment, its effect on MTT reduction was analyzed as described above.

### 2.7. In Vitro Determination of Solar Photoprotection Factor

The solar photoprotection factor (SPF) was determined in vitro according to Mansur et al. [29]. The extracts were used at the concentrations 100, 250, and 500 μg/mL and it diluted in absolute ethyl alcohol PA grade. Several readings were taken between wavelengths 290 and 320 nm. For the blank only the absolute ethyl alcohol PA grade was used. The values obtained were used in the following equation:

Solar Protection Factor (SPF) Spectrophotometric = FC.Σ EE (*λ*) l (*λ*) Abs (*λ*), where FC = correction factor (10), referring to an SPF = 4; EE (*λ*) = erythemogenic effect of radiation; I (*λ*) = intensity of the sun; abs (*λ*) = spectrophotometric reading of the absorbance of the plant extracts. This experiment was done in triplicate.

### 2.8. Statistical Analysis

Statistical analyses were done using the Graphpad Prisma 6 software (Graphpad Software Inc., San Diego, CA, USA) in triplicates using ANOVA. Each experiment was run at least three times. The Tukey’s test was used to compare the differences between extracts. Differences were considered significant when the value of *p* was ≤ 0.001.

## 3. Results

### 3.1. Phytochemical Screening

In this study, a serial extraction approach, from non-polar to polar solvents, was used to identify secondary metabolites. Water comprised 96.6% of the pulp weight of the *M. zehntneri*, which belongs to the Cactaceae family. The amounts of sugars, phenolic compounds, and proteins were measured. The sugar contents of these extracts were as follows: HE, 22.18 µg/mL; CE, 46.83 µg/mL; EE, 107.97 µg/mL; ME, 101.97 µg/mL; FWE, 8.36 µg/mL; and WE, 42.73 µg/mL. The highest amounts of sugar were detected in EE and ME; thus, the order of sugar content was as follows: FWE < HE < WE < CE < ME < EE. Using TLC, we detected the presence of fructose in EE, ME, and WE; sucrose in EE, ME, FWE, and WE; and galactose in EE, ME, and FWE. Furthermore, uronic and glucoronic acids were also detected in EE, ME, and WE. Raffinose was detected in non-polar extracts (HE Rf 0.9; CE Rf 0.9) and EE (Rf 0.9 and 0.75). Fructose was detected in EE, ME, and WE (all of them with Rf 0.2 and 0.3); sucrose was identified in EE, ME, and WE (Rf 0.3); and galactose in EE, ME, and WE (Rf 0.3).

Protein content was also measured and the order was as follows: ME (7.0 µg/mL) < WE (7.1 µg/mL) < EE (10.3 µg/mL) < FWE (10.9 µg/mL) < HE (20.5 µg/mL) < CE (47 µg/mL). In addition, phenolic compounds were detected in all extracts, and the order of phenolic compound content was as follows: WE (0.3 µg/mL) < ME (0.62 µg/mL) < EE (0.68 µg/mL) < FWE (0.74 µg/mL) < HE (4.08 µg/mL) < CE (7.57 µg/mL). 

Saponin, sugars, and glycoproteins (mobile phase III) were present in the polar extracts, namely EE, ME, FWE, and WE. Because phenolic compounds have been associated with antioxidant activities, we identified the phenolic compounds in the extracts using TLC. Terpenes, such as ursolic and oleanolic acids, were identified in all the extracts using mobile phase I and Retention Factor (Rf) whose value were 0.75. 

### 3.2. In Vitro Antioxidant Assay

**Figure 1:**
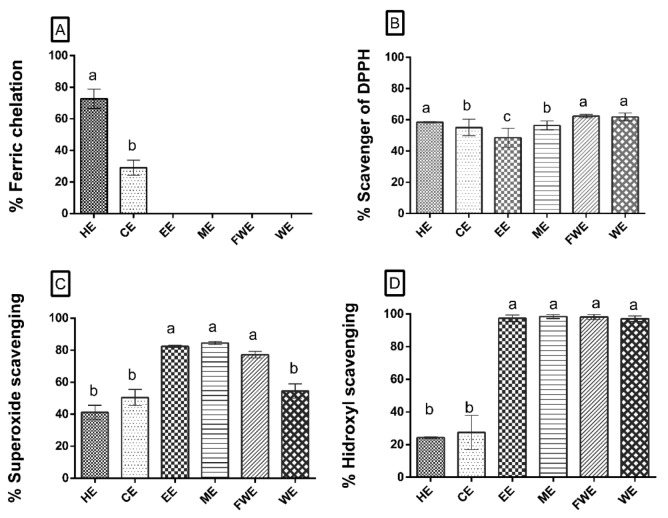
Antioxidant assay. (**A**) Percentage of ferric chelating effect of *M. zehntneri* extracts, where the y-axis corresponds to percentage of ferric ion chelation activity. (**B**) Percentage of DPPH radical-scavenging, where the y-axis corresponds to percentage of DPPH-scavenging activity. (**C**) Percentage of superoxide ion-scavenging, where the y-axis corresponds to percentage of superoxide ion- scavenging activity. (**D**) Percentage of the hydroxyl radical scavenging effect of the extracts at different concentrations, where the y-axis corresponds to percentage of hydroxyl radical-scavenging activity. In these tests, the x-axis corresponds to *M. zehntneri* extracts at a concentration 100 μg/mL for (**A**,**C**,**D**) and at 25 μg/mL for (**B**). The assays were performed in triplicate. HE, hexane extract; CE, chloroform extract; EE, ethanol extract; ME, methanol extract; FWE, final water extract; WE, water extract. The data were analyzed by ANOVA–Tukey test *p* < 0.001. Different letters (a, b, c) correspond to the significant differences in statically analysis.

Considering the presence of phenolic compounds, sugar, and protein in the extract, we analyzed whether these extracts possess antioxidant activity using seven different assays. The antioxidant activities may act in three stages: initiation (TAC (Total Antioxidant Capacity) and DPPH reduction), propagation (ion chelation), and termination (superoxide and hydroxyl radical-scavenging) [30,31]. The extracts were assayed at three different concentrations—100, 250, and 500 µg/mL. Figure 1 shows the results of these assays using 100 µg/mL extracts.

None of the *M. zehntneri* extracts showed total antioxidant capacity (TAC), reducing power activity, or copper chelating activity (data not shown). However, ferric chelating activity was shown by non-polar extracts; HE showed approximately 75% ferric chelating activity at 100 µg/mL, whereas CE exerted 25% activity at 100 µg/mL (Figure 1A). In addition, the iron chelating activity of CE was dose-dependent.

In the DPPH assay, DPPH was saturated and then reduced to a concentration of 25 µg/mL (Figure 1B). DPPH scavenging activity was shown by all extracts, with HE showing approximately 55% activity and the other extracts showing approximately 60% activity. 

EE, ME, and FWE showed the highest superoxide scavenging activity of approximately 80%, whereas HE showed approximately 40% activity, and CE and WE showed approximately 50% activity (Figure 1C). Moreover, the polar extracts showed 100% hydroxyl scavenging activity (Figure 1D), whereas the non-polar extracts (HE and CE), showed approximately 20% activity (Figure 1D).

### 3.3. Biofilm Assay

Considering the antioxidant activity of the *M. zehntneri* extracts, we tested whether these extracts possess anti-biofilm activity against *S. epidermidis* 70D MRSE. In folk medicine, the pulp of *M. zehntneri* is used to treat respiratory tract disorders; therefore, we analyzed the activity of the extract on *S. epidermidis* 70D MRSE. This bacterium is present on the skin, but can also grow easily in medical devices. Furthermore, this bacterium has been reported to be resistant to various antibiotics, posing difficulties in treating its infection [14]. Thus, we determined the MIC and MBC of the extracts against *S. epidermidis* 70D MRSE. The MBC represented the lowest extract concentration that exerted bactericidal action against *S. epidermidis* 70D MRSE. The highest MBC (50 mg/mL) was shown by HE and FWE, whereas for the other extracts the MBC values were >50 mg/mL and >25 mg/mL, respectively. The MIC of CE and WE was 25 mg/mL, whereas their MBCs were 25 mg/mL and >50 mg/mL, respectively. The lowest MIC (6.25 mg/mL) was shown by EE and ME; both had an MBC 12.5 mg/mL.

**Figure 2:**
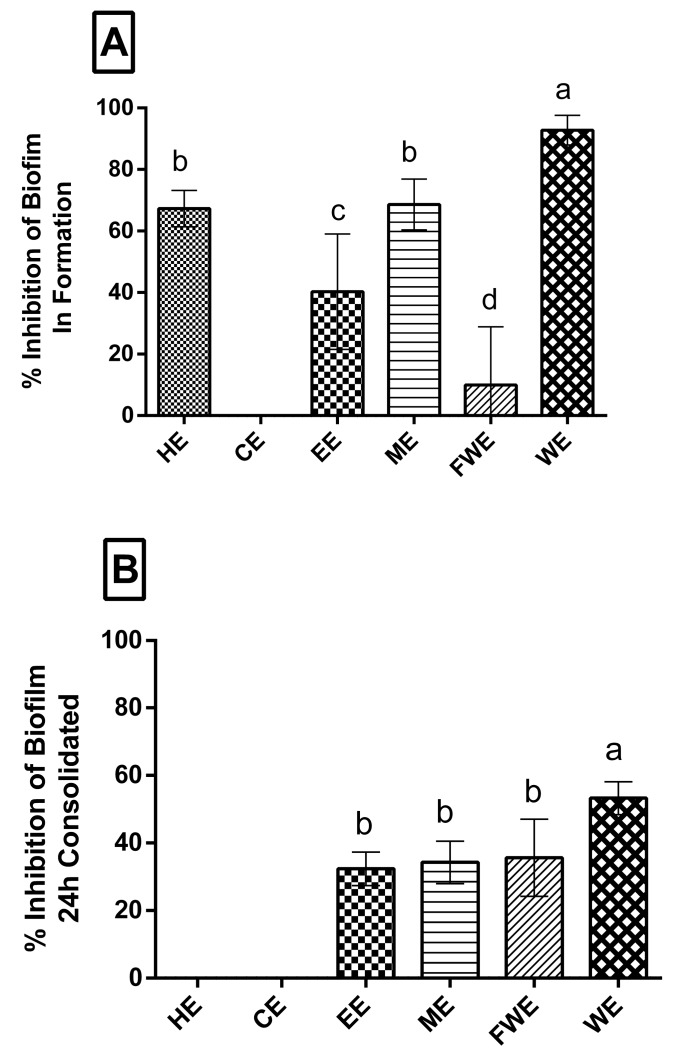
Percentage of biofilm inhibition. Graphs showing the results of the effects of *M. zehntneri* extracts on biofilm formation (**A**) and consolidation (**B**) of *S. epidermidis*, where 100% is the maximum inhibition shown by gentamicin, a positive control. The x-axis represents the extracts and the y-axis represents percentage of biofilm inhibition. This assay was performed in triplicate. HE, hexane extract; CE, chloroform extract; EE, ethanol extract; ME, methanol extract; FWE, final water extract; WE, water extract. Data were analyzed by ANOVA-Tukey test, *p* < 0.001. Different letters (a, b, c, d) correspond to the significant differences in statically analysis.

Next, we investigated biofilm formation and consolidation of *S. epidermidis* 70D MRSE, as it can grow in medical devices, which has a considerable impact on hospitalized patients. We also evaluated whether the *M. zehntneri* extracts affect biofilm formation (Figure 2A) and consolidation (Figure 2B). Figure 2A shows that the extracts affect biofilm formation, with WE showing 40–90% growth inhibitory activity. CE showed no activity, whereas FWE showed a low activity of 10% (Figure 2A). A 30% inhibition of biofilm consolidation was shown by the polar extracts EE and ME, whereas WE showed approximately 50% inhibition. On the contrary, the non-polar extracts (HE and CE) did not have any effect on biofilm consolidation (Figure 2B). 

### 3.4. Cell Viability

#### 3.4.1. MTT Assay

The data presented above revealed that the *M. zehntneri* extracts exhibited antioxidant and antimicrobial activities. Thus, it was important to verify whether these extracts exert any cytotoxicity, which was analyzed using fibroblast cell line, 3T3. Cell viability was measured by MTT assay, with the maximum pattern cell activity occurring at 100% of MTT reduction.

**Figure 3:**
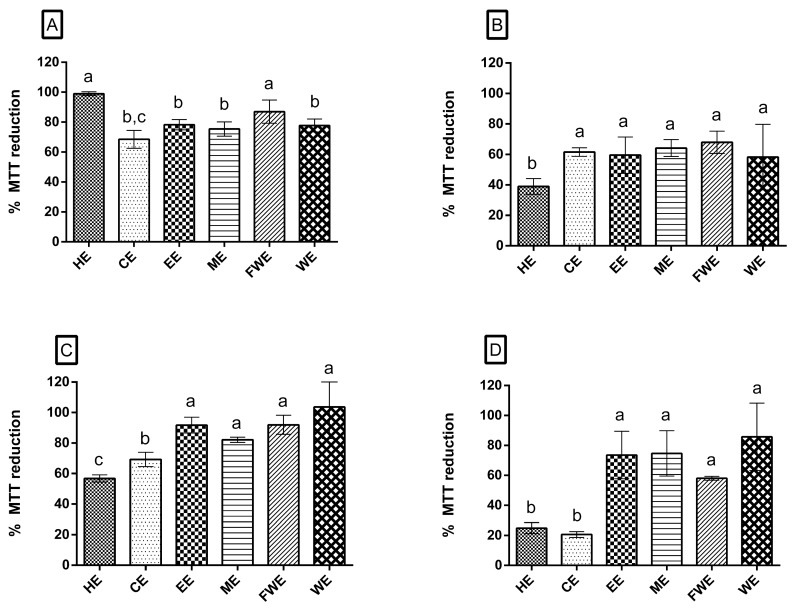
Cell viability. (**A**) Percentage of MTT reduction in 3T3 cells after treatment with *M. zehntneri* extracts at 250 µg/mL. (**B**–**D**) MTT reduction percentage in 3T3 cells after treatment with 250 µg/mL *M. zehntneri* extracts and 0.1 M H_2_O_2_. (**B**) Preventive effect of *M. zehntneri* extracts on H_2_O_2_-treated 3T3 cells. (**C**) Curative effect of *M. zehntneri* extracts on H_2_O_2_-treated 3T3 cells. (**D**) Concurrent effect of *M. zehntneri* extracts on H_2_O_2_-treated 3T3 cells. The x-axis corresponds to *M. zehntneri* extracts at 250 μg/mL and the y-axis corresponds to percentage of MTT reduction. This assay was performed in triplicate. HE, hexane extract; CE, chloroform extract; EE, ethanol extract; ME, methanol extract; FWE, final water extract; WE, water extract. Results were analyzed by ANOVA-Tukey test, *p* < 0.001. Different letters (a, b, c) correspond to the significant differences in statically analysis.

The results of the MTT assay of the extracts at a concentration of 250 µg/mL are presented in Figure 3. Figure 3A shows that HE and FWE had no effect on cell viability (cell viability, 100%), whereas EE, ME, and WE showed a slight effect on cell viability (cell viability, approximately 80%). Cytotoxicity was exhibited only by CE, as it reduced the cell viability to 68% (Figure 3A).

#### 3.4.2. Oxidative Stress

We also evaluated whether the *M. zehntneri* extracts affected the 3T3 cells in the presence of H_2_O_2_. We first determined the concentration of H_2_O_2_ that reduced 3T3 cell viability by 50% (IC_50_), which was assessed to be 0.1 M. Next, we evaluated the preventive (Figure 3B), curative (Figure 3C), and concurrent (Figure 3D) actions of the extracts on H_2_O_2_-treated 3T3 cells.

Five extracts (CE, EE, ME, FWE, WE) showed preventive action, with only HE reducing the cell viability below 50% (Figure 3B). All the extracts showed curative action, showing cell viability of more than 60%. The polar extracts increased cell viability up to 80–100% (Figure 3C). The order of curative activity of the extracts was HE < CE < ME < EE = FWE < WE. HE and CE (non-polar extracts) showed no concurrent action, showing cell viability of lower than 25%. On the contrary, WE, EE, and ME showed concurrent action with cell viability of approximately 75%, whereas EAF showed approximately 60% cell viability. The order of concurrent action of the extracts was FWE < EE = ME < WE. Taken together, the polar extracts had preventive, curative, and concurrent actions on H_2_O_2_-treated 3T3 cells, increasing their viability.

### 3.5. Solar Protection Factor (SPF)

**Figure 4:**
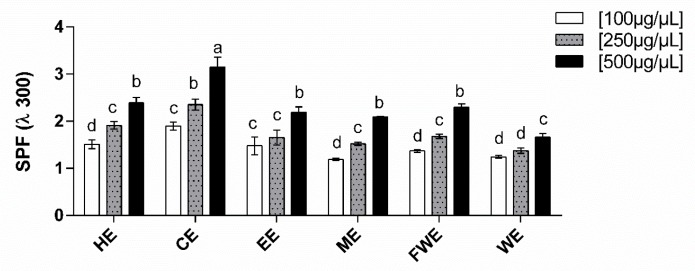
In vitro *solar* protection factor (SPF) from *M. zehntneri* extracts. The x-axis corresponds to *M. zehntneri* extracts at different concentrations; the y-axis corresponds to SPF values obtained at 300 *λ*. This assay was performed in triplicate. HE, hexane extract; CE, chloroform extract; EE, ethanol extract; ME, methanol extract; FWE, final water extract; WE, water extract. Data were analyzed by ANOVA–Tukey test, *p* < 0.001. Different letters (a, b, c, d) correspond to the significant differences in statically analysis.

As secondary plant metabolites have been associated with UV protection, we evaluated whether the *M. zehntneri* extracts have solar protection potential. The solar protection potential of the extracts was dose-dependent; the obtained SPF values increased as the extract concentration increased (Figure 4). The polar extracts, EE, ME, FWE, and WE, had SPF values between 1.2 and 2.3, whereas the non-polar extracts, HE and CE, had SPF values between 2.4 and 3.2, respectively, with CE showing the highest value.

## 4. Discussion

Medicinal plants have attracted increasing attention owing to their importance in traditional medicine as well as in the identification of new molecules with potential for treating various diseases [32]. Several diseases, such as cancer, diabetes, and neurodegenerative disorders including Parkinson’s and Alzheimer’s disease, have been associated with imbalance in oxidative homeostasis; therefore, medicinal plants are important in pharmacological research [33].

Cactaceae plants form important food sources with numerous health benefits, and are used in traditional medicine [34]. Moreover, these plants are known to contain a considerable amount of water, sugar, and organic acids [35].

TLC data showed the presence of terpenes (ursolic and oleanolic acids) in the non-polar extracts. Moreover, saponins, sugars, and glycoproteins were identified in the polar extracts, exposed at 3.1 issue. Ursolic acid is a triterpenoid associated with antiproliferative, anti-inflammatory, and apoptosis-inducing activities, as well as with management of neurodegenerative diseases [36,37]. Further, saponins have been associated with a protective effect against oxidative stress in neurons, exhibiting neuroprotective action by inhibiting Aβ-amyloid and by suppressing lipid peroxide accumulation and GSH reduction on mice hippocampus induced by Aβ_25–35_ protein [7,38].

Moreover, analysis of the TLC data for sugars showed the presence of raffinose in the non-polar extracts, as well as fructose, sucrose, and galactose in the polar extracts. Arabinogalactans, polysaccharides comprised of galactose, arabinose, ramose, and galacturonic acid, have been identified in other Cactaceae plants [39]. Furthermore, raffinose is comprised of galactose, fructose, and glucose (monosaccharides). Thus, polysaccharides (or different combination of monosaccharides) may be considered natural antioxidant polymers [20,40].

As many diseases and neurodegenerative disorders have been associated with ROS imbalance, we used seven antioxidant assays to investigate the antioxidant potential of the extracts. The data obtained showed that the non-polar extracts had ferric chelating activity (Figure 1A). Ferrous turnover is an important process in patients with thalassemia, cardiac disorders, and neurodegenerative diseases; thus, detection of ferric chelating activity in the extracts was a significant finding [41]. Moreover, *M. zehntneri* polar extracts (EE, ME, FWE, and WE) had superoxide radical-, hydroxyl radical-, and DPPH-scavenging activities, (Figure 1B–D). Numerous studies have shown the antioxidant activities of cactus pears. Furthermore, these studies showed that diet supplementation with the pulp of these cactus pears also reduces oxidative stress in patients [42].

It has been proposed that antioxidant activity may be associated with polyphenols, flavonoids, and polysaccharides [20,40,41,43]. Thus, our findings showed that *M. zehntneri* extracts, similar to other cactus extracts, exerted their activity in three stages: initiation (DPPH-scavenging activity, shown by all extracts), propagation (ferrous-chelating activity, shown by the non-polar extracts HE and CE), and termination (superoxide and hydroxyl radical-scavenging activity, shown by the polar extracts EE, ME, FWE, and WE) [31,44].

Because antioxidant activity may be associated with anti-inflammatory and antimicrobial activities [20,44,45], we evaluated the antimicrobial activity of the extracts and their effect on biofilm formation and consolidation. *S. epidermidis* (Figure 2A,B), which normally colonizes the human skin, can affect the balance of microbiota and enter the bloodstream via medical devices, eventually becoming pathogenic [14]. Biofilms are sites where bacterial growth occurs as structured aggregates; these structures confer protection against adverse situations, leading to resistance to antibiotic therapies and immunological elimination. Non-polar (EH) and polar extracts (EE, ME, WE) showed negative effects against antibiofilm formation, which may be related to the presence of triterpenes and saponins, compounds that are known to have antimicrobial activity [46]. Ethanol extracts from *Terminalia fagifolia* also showed antimicrobial activity, which has been attributed to glycosylated and non-glycosylated terpenes and saponins [47]. Furthermore, the activity presented here may also be associated with the combination with polysaccharides, which may change cellular membrane potential or hydrophobicity potential [48]. Additionally, aqueous extracts of *M. zehntneri* prepared using different methods showed antibiofilm potential against the planktonic *S. epidermidis* [8]. These data reinforced the results of our study, which used a pathogenic strain known to form biofilms (*S. epidermidis*) and lower extract concentration. Moreover, Blando et al. [49] showed the antimicrobial potential of *Opuntia ficus-indica* extracts, which was attributed to the presence of monosaccharaides (galactose and glucose).

Other cactus (*O. ficus-indica* and *Pereskia bleo*) extracts showed no cytotoxicity on normal NIH-3T3 cells [50]. These results support our findings that *M. zehntneri* extracts exhibited no cytotoxicity, but showed curative potential. Furthermore, we observed that the extracts induced recovery of 3T3 cells at 30 min following oxidative treatment with hydrogen peroxide (an oxidative stressor agent) (Figure 3). Radad et al. [51] proposed that ginsenoside, a saponin, exerts neuroprotective action against oxidative stress induced by hydrogen peroxide treatment. These data support the possibility that *M. zehntneri* extracts contain compound(s) that can protect 3T3 cells against the oxidative effect of hydrogen peroxide.

Related to the antioxidant potential, the presence of compounds with sun protection effects was also analyzed in the extracts (Figure 4). UVA and UVB radiation have an important effect on skin cells and have been correlated with tumorigenesis [52]. Compounds with solar protection effects may be inorganic or organic and, in general, are inert particles that reflect photons, e.g., zinc oxide or titanium dioxide. The organic compounds are derived from plant secondary metabolites, such as carbonyl groups, polyphenols, and glycosides [52]. To reduce the toxic effects of sunscreen, especially in children, new organic molecules that can be incorporated into sunscreen formulations are required. Wagemaker et al. [53] showed that extracts from coffee species has SPF values of 0.0 to 4.1, and could potentially be used a sunscreen material. The non-polar *M. zehntneri* extracts (HE and CE) at 500 µg/mL showed SPF values of 2.4 to 3.2. Moreover, Saewan et al. [54] proposed that to develop new sunscreen products with protective effects against UV radiation, it is possible to use different strategies, such as combinations of organic and inorganic molecules.

## 5. Conclusions

These results evoke interest in obtaining new molecules from natural sources for the development of therapeutic products with various functions. We showed the in vitro antioxidant activity, anti-biofilm potential, cell viability, cell curative potential, and solar protection effects of *M. zehntneri* extracts. These activities may be attributable to a single molecule or to the synergy between two or more molecules present in these extracts. Further studies need to be conducted to improve our understanding of these molecules and their activities.
